# Lignin-Based Composite Materials for Photocatalysis and Photovoltaics

**DOI:** 10.1007/s41061-018-0198-z

**Published:** 2018-05-02

**Authors:** Ayesha Khan, Vaishakh Nair, Juan Carlos Colmenares, Roger Gläser

**Affiliations:** 1 Institute of Physical Chemistry, Polish Academy of Sciences, Kasprzaka 44/52, 01-224 Warsaw, Poland; 2 Institute of Chemical Technology, Leipzig University, Linnéstr. 3, 04103 Leipzig, Germany

**Keywords:** Lignin, Composite materials, Photocatalysis, Photodegradation, Photovoltaics, Photoactive materials

## Abstract

Depleting conventional fuel reserves has prompted the demand for the exploration of renewable resources. Biomass is a widely available renewable resource that can be valorized to produce fuels, chemicals, and materials. Among all the fractions of biomass, lignin has been underutilized. Due to its complex structure, recalcitrant nature, and heterogeneity, its valorization is relatively challenging. This review focuses on the utilization of lignin for the preparation of composite materials and their application in the field of photocatalysis and photovoltaics. Lignin can be used as a photocatalyst support for its potential application in photodegradation of contaminants. The interaction between the components in hybrid photocatalysts plays a significant role in determining the photocatalytic performance. The application of lignin as a photocatalyst support tends to control the size of the particles and allows uniform distribution of the particles that influence the characteristics of the photocatalyst. Lignin as a semiconductive polymer dopant for photoanodes in photovoltaic cells can improve the photoconversion efficiency of the cell. Recent success in the development of lignosulfonates dopant for hole transport materials in photovoltaics will pave the way for further research in lignin-based high-performance organic electronic devices.

## Introduction

The chemical industry principally depends on fossil resources for the manufacturing of carbon-based compounds. However, dwindling supply of conventional fuels and the search for alternative raw materials for chemical production has made biomass an attractive resource that has significant potential for the production of chemicals, fuels, and materials, paving the way for a sustainable future [1]. Lignin is a major fraction of biomass besides cellulose and hemicellulose that accounts for 40% of the total lignocellulosic biomass energy. However, little attention has been paid to the valorization of lignin due to its complex nature [2]. Recently, works have been reported for the conversion of lignin into value-added chemicals like aromatics [3], low molecular weight hydrocarbons, and fuel [4] through depolymerization reactions and gasification, respectively.

The term “lignin” is devised from the Latin word “lignum”, which means wood [5]. Lignin accounts for about 15–30% of the total biomass content in plants, annually about 150 billion tons of lignin is produced by plants, which make it the most abundantly available natural polymer next to cellulose. It stores about 0.082% (3000 EJ year^−1^) of all the solar radiation intercepted the earth surface, which accounts for approximately 5.4 times the present global energy consumption rate. With the empirical formula of C_31_H_34_O_11_, lignin contains about 95 billion tons of the carbon in the earth crust, which illustrates the unexploited high carbon energy reserve [6].

**Fig. 1:**
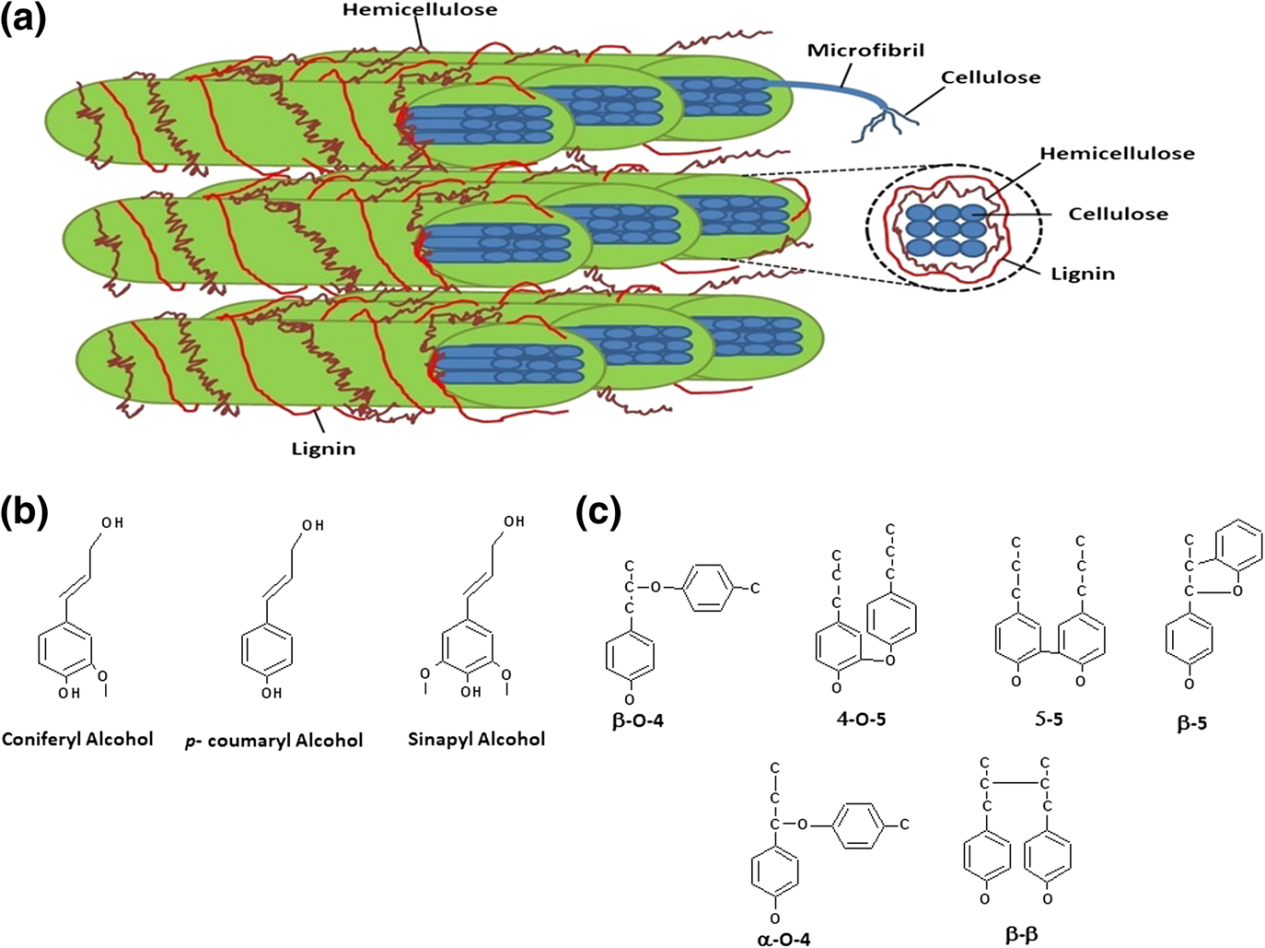
**a** Plant cell wall structure and microfibril cross section (strands of cellulose molecules embedded in a matrix of hemicellulose and lignin) [12]. **b** Monolignols units in lignin [12]. **c** Common linkages found in lignin [13]

Lignin has an extensively branched three-dimensional chemical structure with various functional moieties such as carboxyl (COOH), carbonyl (C=O), and methoxy (CH_3_O), respectively. It is a macromolecule made up of repeating phenyl propane-based monolignols subunits, which are coniferyl alcohol (G), sinapyl alcohol (S), and low amounts of *p*-coumaryl alcohol (H) that take part in lignin formation (Fig. 1). Common linkages found in heterogeneous, high molecular weight lignin are β-O-4, α-O-4, β-5, β–β, 5-5′, 4-O-5, β-1′ [7]. The percentage content of monolignol subunits varies among different plant species. Similar to the monomers content, there is also a variation in the percentage of linkages with respect to plant species [8]. The variation in linkages and monolignols content with plant species make the actual structure determination of lignin rather difficult [9].

Among all the fractions of biomass, lignin has been comparatively underutilized attributed to its complex structure, recalcitrant nature, and heterogeneity that make its valorization relatively challenging. Lignin comprises 30% of all the organic carbon stockpiled in the biosphere [10, 11]. Moreover, the pulp and paper as well as the bioethanol industry produce copious amounts of lignin as a side product that is mainly exploited for power and heat generation via combustion. Out of 50 million tons of lignin produced by the pulp and paper industry in 2010, only 2% has been utilized for the production of chemicals, while a high fraction is just burned as low-quality fuel [9]. However, the high aromaticity of lignin makes it a potential precursor for the production of a number of chemicals. In the context of sustainability and economic viability, lignin valorization may play a key role. Therefore, there is a need to concentrate on developing efficient, cost-effective, and green methods for the valorization of lignin [11].

Along with the generation of value-added chemicals, application of lignin for manufacturing of high-performance materials with tunable structure and properties such as high-value polymeric materials or supercapacitors to adsorbents and from biomedical application to electrode materials [14, 15] is progressively becoming a focus of current research [9]. In 2014, the overall lignin market around the globe was valued at approximately US$775 million, and it is anticipated that in 2020 it will reach approximately US$900 million, consistent with an annual average growth rate of 2.5% within 2015 and 2020 [16]. A moderately slow growth rate originated from the impediments in handling of lignin due to its complex nature as well as from the isolation methods employed that utilizes lignin as a fuel [17].

The basic aim of this review is to summarize the recent progress in lignin-based materials for their application in heterogeneous photocatalysis (2013–2017) and photovoltaics (2015–2016). Photocatalysis and photovoltaics are the two major routes for the exploitation of solar energy. Moreover, photocatalysis is a key route, specifically for the degradation of contaminants in water. Recently, some lignin-based composites have shown high activity for the photocatalytic environmental remediation processes such as desulphurization and degradation of organic dyes [18–20]. Organic photovoltaic cells have gained much attention over the last few decades due to their potential application in reducing energy and environmental impact caused by the increasing combustion of fossil fuels. Substantial efforts have been made towards understanding the mechanism of photovoltaic cells together with modifying chemical structural motifs and device structure leading to the enhancement of power conversion efficiency from 4% to over 25% using silicon. Although the energy conversion efficiency of the conventional materials like crystalline Si is high (25%), these materials also have high manufacturing and installation costs [21]. Another critical aspect in photovoltaic devices is the anode interface, where hole extraction and hole injection takes place; a hole conducting polymer is required for this function. PEDOT:PSS is most commonly used conducting polymer applied in anode interfaces. However, it has several drawbacks such as its acidic nature that induces corrosion and variable conductivities due to its microstructural and electrical inhomogeneity. Lignosulphonates have recently been introduced as dopant with tunable conductivities and work function to modify the anode interface [22]. Application of lignin in the field of photovoltaics for the development of organic electronic devices is a sustainable approach towards electricity generation. Nevertheless, several articles have been published on carbon-based hybrid materials for photocatalysis, specifically on the degradation of organic contaminants in water [23], but insufficient information is available on the synthesis of lignin-based composite materials and their limitations and applications in the field of photocatalysis and photovoltaics.

## Native Versus Processed Lignin

### Sources

Lignocellulosic biomass is the natural source of lignin. Both woody and non-woody biomass resources contain significant amounts of lignin [24]. Lignin along with cellulose and hemicellulose is a principal constituent of a plant’s cell wall. The key function of lignin in the cell wall is to provide rigidity by reinforcing strength of crystalline cellulose and middle lamella that enable the erect growth of plants [9]. The content of lignin varies considerably among different plant species, and typically decreases from softwood to hardwood to grasses [24]. Furthermore, approximately 50 million tons of lignin is produced annually by chemical processing of pulp in bioethanol refineries and paper industry [17, 25].

### Classification of Lignin

Lignin is categorized into the following three major classes based on its origin [24]

- Guaiacyl lignin contains significantly high concentrations of coniferyl alcohol with the G:S:H ratio of 90:2:8. It is also named softwood lignin, mainly derived from coniferous trees.
- Guaiacyl–syringyl lignin contains significant amounts of sinapyl alcohol in addition to coniferyl alcohol. It is also known as hardwood lignin, principally found in deciduous trees and shrubs.
- Guaiacyl syringyl *p*-hydroxybenzaldehyde lignin contains a significant proportion of *p*-hydroxybenzaldehyde, approximately 30% in combination with other phenyl propane subunits. Lignin derived from monocotyledons falls into this category.

### Extraction of Lignin

The isolation of lignin from lignocellulosic biomass is carried out under different conditions where polymeric lignin is chemically degraded to low molecular weight fragments, with different physicochemical properties. In addition to the source, composition and properties of the isolated lignin vary depending on the method of extraction. Generally, acid- or base-catalyzed reactions are commonly used in the extraction and depolymerization of lignin [24]. At an industrial scale, four methods are generally employed for lignin extraction that can be further categorized into two classes based on the presence of sulfur. Sulphur-containing lignin is extracted through the sulfite and Kraft processes. On the other hand, soda and organosolv processes are applied for the isolation of non-sulphur-containing lignin [9].

#### Sulfite Process

Sulfite-pulping process is a commonly used method for the production of commercial lignin. Different concentrations of sulfite or bisulfite salts of ammonium, magnesium, sodium, or calcium are used in an aqueous solution within the pH range of 1–13.5 [26]. The reaction temperature is usually maintained between 140 and 160 °C [27]. Delignification in sulfite pulping process involves the sulfonation of the aliphatic chain of the lignin via cleavage of α-O-4-ether. The lignosulfonates produced through the sulfite process are water soluble and easily dissolved in pulping liquor in an aqueous media [9].

#### Soda Process

The first pulping process applied for the isolation of lignin was the soda process, introduced by Watt and Burgess in 1854 [28]. The soda process involves heating of the biomass in an alkaline aqueous solution (sodium hydroxide solution) at a temperature of around 160 °C [9]. The reaction proceeds with the protonation of phenolic hydroxyl moieties of lignin with simultaneous cleavage of α-O-4 and β-O-4 bonds [17]. The lignin produced through the soda process is soluble in water and upon acidification it is isolated from pulp liquor through precipitation reaction [9].

#### Kraft Process

The most commonly used method for pulping is the Kraft process, which produces sulphur-containing lignin. Large fractions (98%) of lignin produced through the Kraft process are utilized for energy purposes through combustion, and merely minor fractions (2%) are used for material or chemical synthesis [9, 17]. The Kraft process is supposed to be an advancement or progression of the soda process, as it involves the heating of pulping liquor with sodium sulfide in addition to sodium hydroxide at a temperature between 150 and 180 °C [9, 26]. The process of lignin depolymerization in the Kraft process is the same as in the soda process; the reaction proceed with the cleavage of α-O-4 and β-O-4 ether linkages and results in soluble fragments of lignin [17]. A minor fraction of resultant lignin is sulphated due to the presence of anions of hydrosulfide. A large fraction of lignin produced is sulphate-free and isolated via acidification and precipitation method [9, 29].

#### Organosolv Process

The most recent process used for the extraction of lignin at an industrial scale is the organosolv process. This technique involves heating the biomass with a mixture of organic solvents for isolating lignin. Polar organic solvents such as methanol, acetone, ethanol, acetic acid, and formic acid are commonly used for the extraction process [9, 17]. The nature of the solvent used significantly determines the structure and properties of the isolated lignin [9].

### Properties of Lignin

Native lignin is colorless, but acid–alkali treatment changes its color to dark brown [5]. Physical and chemical properties of the lignin vary with the extraction procedure and the monolignols content [9], as shown in Table 1. The functional moieties of lignin like carboxylic, phenolic hydroxyls, methoxy, aliphatic and carbonyl groups depend on the monomeric linkages. Moreover, these moieties significantly contribute to the chemical modification of lignin [9]. The molecular weight of lignin ranges between 1000 and 20,000 g mol^−1^. As lignin constantly degrades during the extraction process, it is therefore difficult to predict the degree of polymerization attributed to random repetition of subunits [25].

**Table 1:** Summary of characteristic properties of lignin

Type of lignin	Kraft	Lignosulfonate	Soda	Organosolv	References
Structure					[80]
Separation methods	Precipitation (pH change)–ultrafiltration	Ultrafiltration	Precipitation (pH change)–ultrafiltration	Precipitation (addition of non-solvent) Dissolved air flotation	[80]
Active extracting agent	NaOH, Na_2_S	H^+^ , HSO_3_^−^	NaOH	40–60 wt% aqueous ethanol	[26, 111]
pH of isolation medium	13–14	1–2 (acid bisulfite) 3–5 (bisulfite)	13–14	–	[26, 111]
Temp. (°C)	155–175	125–145 (acid bisulfite) 150–170 (bisulfite)	155–175	180–210	[26, 111]
Sulphur (%)	1.0–3.0	3.5–8.0	0	0	[80]
Nitrogen (%)	0.05	0.02	0.2–1.0	0–0.3	[80]
Molecular weight (× 10^3^ g mol^−1^)	1.5–5 (up to 25)	1–50 (up to 150)	0.8–3 (up to 15)	0.5–5	[80]
Solubility	Alkali and some organic solvents	Water	Alkali	Broad range of organic solvents	[80]
*T*_g_ (°C)	140–150	130	140	90–110	[80]
*T*_d_ (°C)	340–370	250–260	360–370	390–400	[80]
Scale (ktpa)	60	1000	5–10	∼ 3	[112]

The glass transition temperature (*T*_g_) of lignin differs with moisture content, molecular weight, extraction method, cross-link density, and measurement method. *T*_g_ generally increases with the increase in molecular weight, based on the structure and fragments’ molecular mass the *T*_g_ of lignin falls between 70 and 170 °C [30]. Analogous discrepancies have been observed for the decomposition temperature of lignin, lignin source, extraction method, and measurement techniques influence lignin decomposition processes [31]. Distinct decomposition of lignin comparable to other biomass components have been observed in the range of 360–480 °C via thermogravimetry [32].

Furthermore, lignin has many valuable properties such as antimicrobial and antioxidant nature [33], mechano-thermal stability [34], and blending properties [5] that make it a potential candidate as a constituent of composites. In addition to its benefits, it has some limitations, such as abrupt changes in its properties with change in moisture content. Bleaching of produced radicals and reaction with atmospheric oxygen is the major drawback of lignin for its application in material synthesis [35]. These limitations may be overcome by chemical modification introduction of lignin into composite materials [36].

## General Applications of Lignin

### Synthesis of Materials from Lignin

Depletion of fossil resources and associated environmental problems has resulted in exploration of renewable resources for the synthesis of materials. Lignin as a renewable biopolymer has a big role to play in this field due its natural abundance. Lignin is a potential candidate for the fabrication of composite materials credited to it several remarkable features such as biodegradability, antioxidant activity, antimicrobial activity, and reinforcing properties etc. [37]. Recently composites based on lignin gained a lot of attention due to its property to be used as a reinforcing material for the synthesis of high-performance composites. Morandim-Giannetti et al. investigated the application of lignin as additive for the fabrication of polypropylene–coir composites [38]. The tensile strength of the composite has not been affected to a large extent by the addition of lignin, though initial degradation temperature and oxidation induction time of the composite has been increased due to the presence of lignin. Moreover, the multifunctional nature of lignin makes it a reactive component for the manufacturing of resins and polymer-based materials. This goal is achieved either through chemical modification of lignin through esterification, phenolation and oxypropylation reaction, or through partial substitution of traditional materials by lignin. Similarly, lignin has shown promising application for the preparation of phenol–formaldehyde resins, epoxy resins, polyurethanes, and graft copolymers [17].

Lignosulfonate and kraft lignin in combination with activated carbon have been used in direct carbon fuel cells for the production of electricity [39]. Lignosulfonate showed better performance than Kraft lignin in direct carbon fuel cells due to high hydrophilicity of lignosulfonate. High wettability of lignosulfonate also enhances electrochemical reactivity and electrical conductivity in fuel cells. Using lignosulfonate-activated carbon, the maximum power density reaches 25 mW cm^−2^, while Kraft lignin-activated carbon shows a power density of 12 mW cm^−2^ [40]. Furthermore, a study reported the production of highly nanoporous carbon for supercapacitors application using low-cost renewable lignin as precursor [41]. The better control over the surface functional groups, pore structure, and electrical conductivity of lignin-based carbon materials enhances the electrocapacitive performance of electrode for supercapacitors [14]. However, there are certain challenges associated with the lignin for its use in energy storage devices such as low electrical conductivity that make the active sites of lignin electrochemically inaccessible. The other factor is the solubility of some types of lignin like lignosulfonates in an aqueous media that may result in degradation of the active material in an electrochemical device [42]. Designing hybrid capacitors using lignin in combination with metal oxides and conductive polymers is a futuristic approach to improve the electrode capacitance of supercapacitors.

## Overview of Carbon-Based Materials in Photocatalysis and Photovoltaics

Recently, tremendous attention has been paid to the development of porous carbon-based materials derived from environmentally friendly renewable biomass resources [43]. Carbon-based materials derived from biomass such as wood, cellulose, lignin, hemicellulose, and biochar widely used as template for semiconductors in photocatalytic applications [44]. Introducing carbon-based materials as doping agent plays a significant role in the modification of photocatalyst by improving visible light responsive performance of the photocatalyst [45, 46]. During photocatalysis, the high surface area, electrical conductivity, and porosity of carbon materials may increase the adsorption and modify the mechanism of photochemical reaction. This synergistic effect induced by carbon materials enhances the photocatalytic degradation of environmental pollutants, photocatalytic production of H_2_, and photocurrent generation attributed to their high electroconductivity [47].

Various efforts have been made for the utilization of carbon-based materials like activated carbon and biochar for fabrication of composites. Activated carbon derived from biomass is considered to be a potential support for the photocatalytic material attributed to its ability to improve the interface charge transfer rate and reduce the electron hole recombination rate [48]. It has shown promising properties as support for TiO_2_ in case of gas and water remediation. Moreover, the heterojunction formed between the components leads to inoculation of electrons from activated carbon to TiO_2_ [49]. Similarly, TiO_2_ in combination with biochar (a porous solid-rich byproduct of thermal decomposition of organic waste) derived from Miscanthus straw pellets and soft wood pellets has shown enhanced photocatalytic activity for selective oxidation of methanol to methyl methanoate as well as for phenol degradation [50].

Among various biomass fractions, cellulose has been extensively utilized for the fabrication of hybrid photocatalyst [20, 51]. Composite films of TiO_2_ show high photocatalytic degradation efficiency for concentrated phenol accredited to the void formation in TiO_2_ assembly and their immobilization by hydroxyl groups [52]. Carbon-based photocatalyst composites with well-defined physicochemical characteristics such as specific surface area, pore volume, microstructure, and solubility etc., may enhance the photocatalytic system that ultimately pave the way to understand the reaction mechanism of the material synthesis based on its structure and composition [44]. Still, further investigation is required to understand the reaction kinetics and mechanism, interphase interaction, and leaching of components from composites.

Photovoltaics is another foremost mode for the utilization of solar energy for power generation. Due to high energy demand, there is a dire need to fabricate energy conversion devices utilizing renewable resource in accordance with the principles of green chemistry. About 1.8 × 10^11^ MW power is intercepted by the earth from the sun, which is quite greater than the current rate of overall energy consumption [53]. The field of photovoltaics offers great potential for the utilization of renewable solar power by converting it into electricity. Steady progress has been achieved in the field of photovoltaics in order to increase the power conversion efficiencies and lower the cost of production by using organic molecule or polymers in chemical design [54].

Photovoltaic technologies are required to be economically viable and environmentally friendly. The current photovoltaic devices are mostly centered on the use of toxic and expensive inorganic chemicals [55] such as CdTe, GaAs, CuIn_*x*_Ga_1−*x*_Se_2_ [56]. Semiconductors derived from organic polymer are potential alternative for inorganic semiconductive materials in the field of photovoltaics. Low cost, renewability, and conjugated structure are the most important features of organic polymers for their application in photovoltaics [57].

**Fig. 2:**
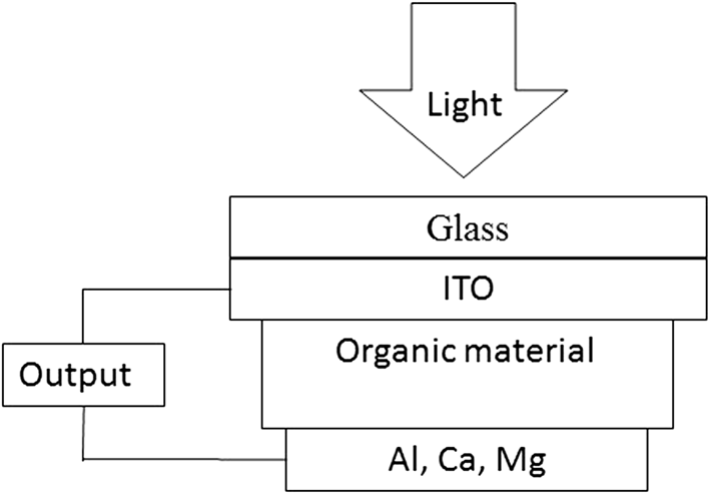
Basic construction of a photovoltaic cell with typical electrode materials. An organic material is sandwiched between two electrodes [57]

**Fig. 3:**
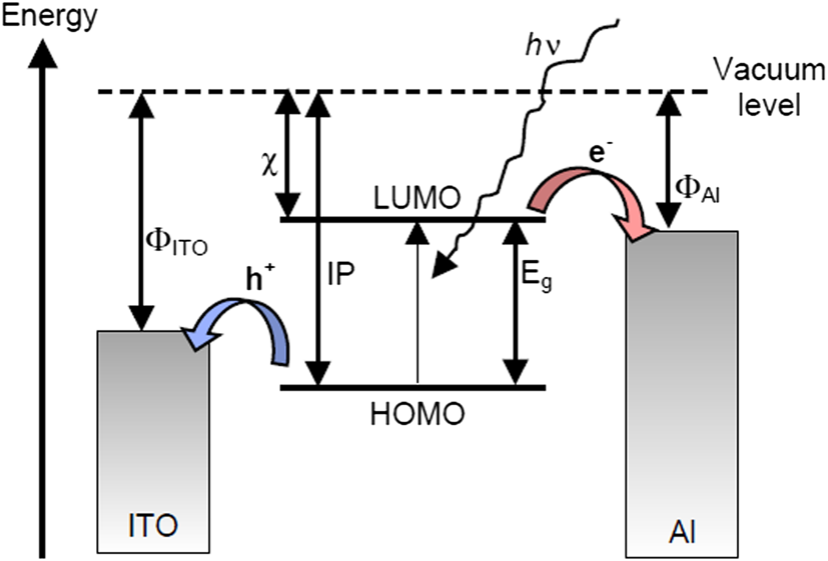
Energy levels and light harvesting. Upon irradiation, an electron is promoted to the LUMO leaving a hole behind in the HOMO. Electrons are collected at the Al electrode and holes at the ITO electrode. *Φ*, workfunction; *χ*, electron affinity; IP, ionization potential; *E*_g_, optical band gap [57]

The importance of natural polymers for the fabrication of photovoltaic devices can be understood by the working principle of photovoltaics. A photovoltaic cell is composed of a layered structure in which the layer that absorbs light is packed between two different types of electrode, as depicted in Fig. 2. One of the electrodes is made up of indium–tin-oxide ITO, while the other electrode is often composed of metals like aluminum, calcium-magnesium, and gold, etc. Upon exposure to light, electrons residing in the highest occupied molecular orbital (HOMO) absorbing a certain wavelength of radiation and shifted to the lowest unoccupied molecular orbital (LUMO) result in the formation of exciton. Free electrons and holes generated through exciton dissociation move towards Al and ITO respectively, as depicted in Fig. 3. Movement of electrons in the external circuit generates an electric current. Asymmetrical electrodes’ ionization energy or work functions provide an electric field required to avoid recombination. This asymmetry is responsible for the flow of electrons from the region of low work function to the region of high work function, the process is known as rectification. In case the semiconductors are based on inorganic materials, interaction of HOMOs and LUMOs of adjacent molecules result in a conduction and valence band. While in case of semiconductors based on organic dyes instead of bands, charge transfer takes place between localized states through hopping. Polymers have a conjugated structure placed in between organic dyes and inorganic semiconductors [57].

Nanocellulose has been used as substrate for the fabrication of photovoltaic and solar cells [58]. Carboxymethylated nanocellulose paper has been applied as a substrate in a device consisting of poly(3,4-ethylenedioxythiophene):poly(styrene sulfonate) (PEDOT:PSS), poly(3-hexylthiophene) (P3HT), and [6]-phenyl-C61-butyric acid methyl ester (PCBM). The photo conversion efficiency of the device was not very high (0.2%), which was attributed to the high resistance of ITO [59].

The dye-sensitized solar cell is a type of photovoltaic cell that works on the principle of oxidation–reduction reaction with the capability of maximizing the power conversion efficiency up to 11% [60]. The microcrystalline cellulose applied as gel electrolyte for the dye-sensitized solar cell. The photovoltaic efficiency of dye-sensitized solar cell based on cellulose gel can be optimized by regulating cellulose concentration and ionic liquid volume ratio. The photoconversion efficiency reaches 3.33% by using a gel composed of LiI (2 wt%), iodine (10 wt% of the whole weight of iodide), microcellulose (5 wt%), 4-*tert*-butylpyridine (10 wt%), and 1-methyl-3-propylimidazolium/1-ethyl-3-methylimidazolium thiocyanate (50/50 volume percentage) when stimulated more than 8 h under solar irradiation [61].

### Lignin-Based Composites in Photocatalysis

#### Preparation Techniques of Lignin-Based Composite Photocatalyst

There are a number of methods employed for the synthesis of photocatalysts with lignin as a support (Table 2). Depositing or immobilizing a photoactive material on or within the pores of lignin-based supports might offer substantial gains in photocatalysis, especially if the photoactive component can be introduced in an oriented or assembled fashion. There are several methods used for the preparation of lignin-based photocatalyst such as solid-phase synthesis, solvent evaporation method, cocalcination, one-pot in situ method, and pH-assisted precipitation (see Table 2 for details).

**Table 2:** Potential use of different types of lignin for the synthesis of composite photocatalyst and their preparation methods

Entry no.	Lignin-based composite photocatalyst	Type of lignin	Method	References
1	Nano-ZnO lignin–amine composite	Aminated lignin	Solid-phase method	[81]
2	SLS–CuO/ZnO nanocomposites	Sodium lignosulphonates (SLS)	Solid-phase grinding method	[68]
3	Lignin@TiO_2_ composites	Kraft lignin Organosolv lignin Low sulfonate content (LSC) Sodium lignin Sodium lignin without sugars Alkali lignin	Solvent evaporation method	[114]
4	Porous carbon–CeO_2_ composites	Sodium lignin sulfonate	Cocalcination method	[20]
5	TiO_2_–lignin composite	Alkali lignin	pH assisted precipitation	[63]
6	Lignin-based carbon/ZnO composite	Alkali lignin	One-pot carbonization method	[18]
7	Lignin-based carbon/ZnO nanocomposite	Alkali lignin	One-pot in situ method	[19]
8	Aminated lignin–CuO nanoparticles	Aminated lignin	Solid-phase method	[70]
9	Lignin–TiO_2_ mixture	Commercial lignin from nonwoody biomasses like wheat straw and sarkanda grass by soda pulping process using aq. NaOH	Ball mill via dry milling and wet milling	[65]
10	Nano TiO_2_–lignin composite	Alkali lignin	Hydrolysis precipitation method	[72]
11	LPQAS–ZnO crystallites	Alkali lignin	One-step precipitation method	[75]
12	SL–ZnO array	Sodium lignosulphonate	Precipitation method	[77]
13	Nano ZnO–AL	Alkali lignin	Solid-state reaction	[113]
14	SLS-functionalized MWNTs/SnO_2_ hybrids SLS-functionalized MWNTs/CdS hybrids	Sodium lignosulfonate	Grinding-in situ formation method	[79]

Precipitation is one of the most frequently applied and cost-efficient methods for the synthesis of photocatalyst composites. More than one layer of photoactive material can be deposited in a homogeneous distribution on the lignin-based support without using costly solvents [62].

**Fig. 4:**
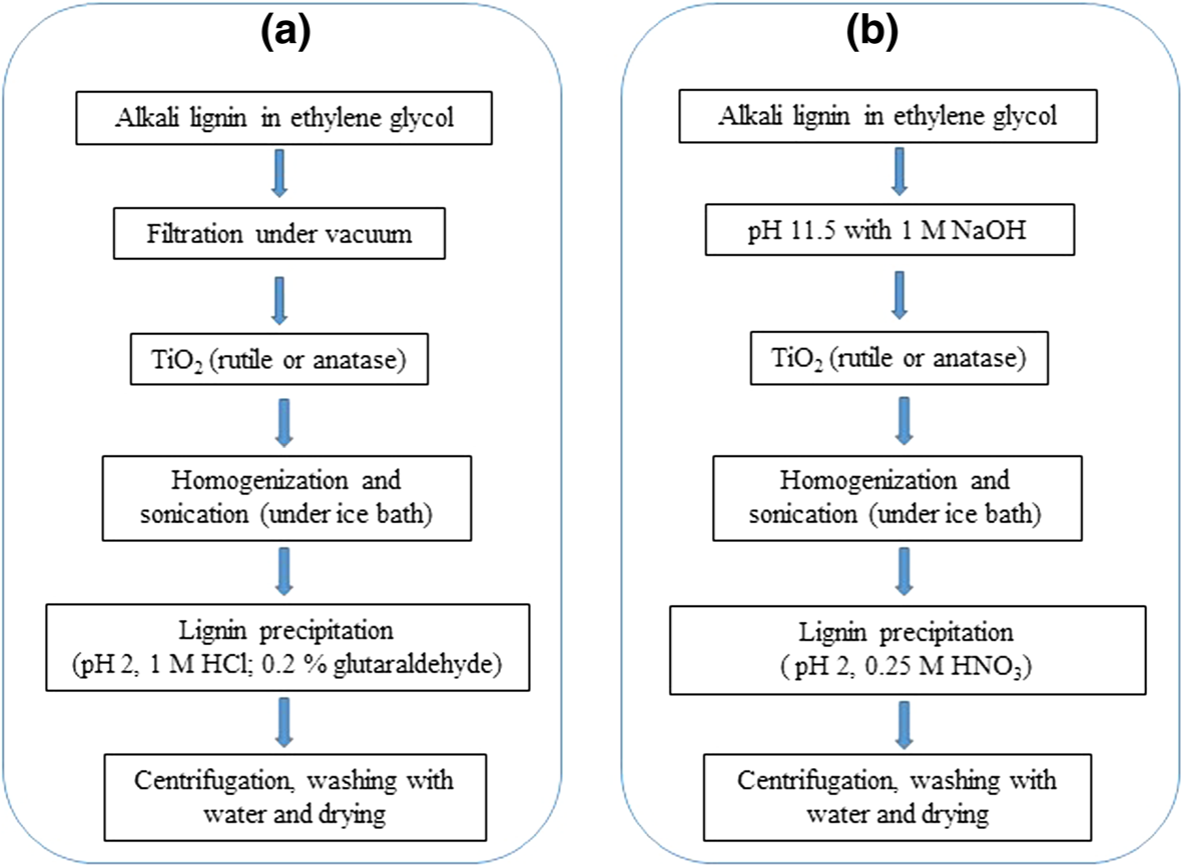
Detailed protocols adopted for the preparation of the lignin/TiO_2_ composite materials [63]

Morsella et al. reported the synthesis of TiO_2_–lignin composite by coating the TiO_2_ nanoparticles with lignin as shell via pH-assisted precipitation (entry 5, Table 2) [63]. The method involves the solubilization of alkali lignin in an alkaline (B) or organic solvent (A) followed by the addition of TiO_2_ particles. The resulting solution is sonicated to ensure homogenization and finally TiO_2_–lignin clusters precipitated out by decreasing the pH of the solution with the help of acids [63]. The detailed scheme for the synthesis of TiO_2_–lignin composite is given in Fig. 4.

Also, facile mechano-chemical processes like ball milling can be employed for the preparation of photocatalyst composites based on lignin [64]. For instance, lignin–TiO_2_ composites were prepared (entry 9, Table 2) through dry and wet milling techniques in a ball mill [65]. In this example, lignin and TiO_2_ with a mass ratio of 1:1 were milled for 6 h at 120 rpm. The obtained dry milled samples undergo wet milling by the addition of different solvents like water, hexane, or acetone with a solvent mass ratio of 1:2. The obtained composites were filtered and dried around 40 °C before application [65].

Furthermore, the one-pot in situ method has also been used for the preparation of composite photocatalysts. In this method, firstly the core photoactive particles are synthesized, followed by the addition of the coating agent or the template precursor. The reaction mixture is stirred and the resulting composite is washed, dried, and calcinated [66]. A recent study reports the synthesis of lignin-based carbon–ZnO nanocomposites using alkali lignin from pulping liquor and zinc nitrate as a precursor for support and ZnO nanoparticles, respectively [19]. The in situ method is considered to be a low-cost and environmentally friendly technique that can use industrial alkali lignin, while one of the drawbacks of the process is the possibility of the entrapment of impurities between the photoactive core and template [19, 66].

Another method used for the preparation of photocatalytic composites is solid-phase grinding. This synthesis approach involves the deposition or attachment of a substrate on a polymer support by grinding and mixing. After the completion of the reaction, the precipitates obtained is repeatedly washed with solvents to remove excess reagents [67]. Among the materials prepared by this method, nanocomposites of CuO–ZnO were synthesized (entry 2, Table 2) using sodium lignosulphonate as the support and zinc carbonate the precursor for the semiconductor component [68]. One of the advantages of solid-phase synthesis is the easy separation of the reactants from final products by washing and filtration [69]. Similarly, Wang et al. reported the synthesis of a CuO nano-photocatalyst based on aminated lignin by the solid-phase technique [70]. CuO particles were obtained through direct reaction of sodium hydroxide and copper nitrate with aminated lignin.

#### Chemical Interaction Between Lignin and Semiconductor

**Fig. 5:**
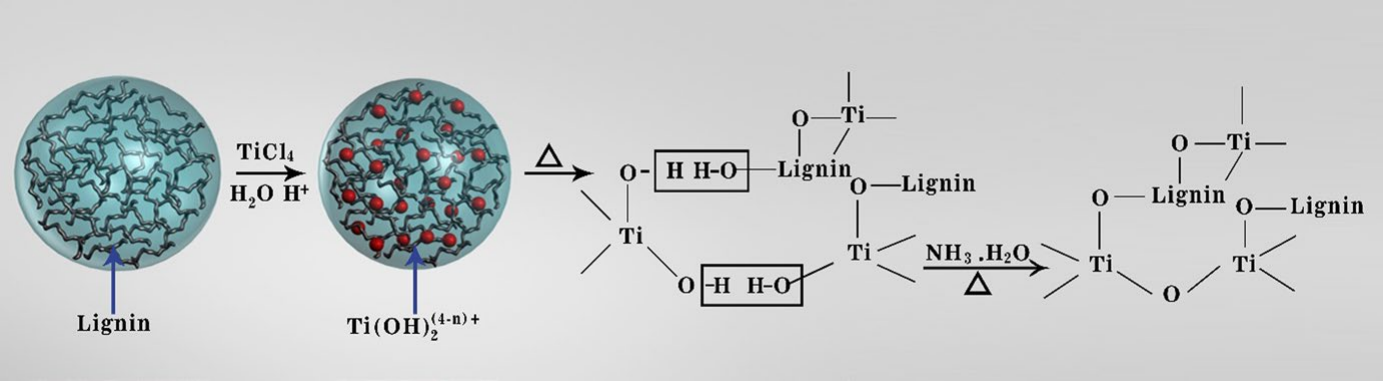
Formation mechanism of mesoporous TiO_2_ with lignin as a template [72]

The hydroxyl groups along with other functional groups in lignin like carboxyl and carbonyl group can engage in specific interactions with the precursors of functional components such as polymers and photocatalysts during the formation of composites [71, 72]. Recently, lignin has been applied as a template to prepare mesoporous TiO_2_ nanoparticles using TiCl_4_ as precursor [72]. The highly electronegative hydroxyl moieties on the surface of the lignin develop a strong affinity towards electropositive metal ions, as shown in Fig. 5. The positively charged Ti(OH)_*n*_^(4−*n*)+^ formed during the partial hydrolysis of TiCl_4_ has affinity for the nucleophilic ligand, resulting in adsorption on the surface of lignin via electrostatic forces of attraction. The adsorbed Ti(OH)_*n*_^(4−*n*)+^ further hydrolyzes and converts to Ti–(O–lignin)_4_ over the surface of the lignin. After complete hydrolysis, well-dispersed TiO_2_ nanoparticles are formed on the surface of the lignin (Fig. 5) [72, 73].

Amine groups can modify the surface of lignin consequently, increasing the molecular weight as well as the number of active groups on the surface. Synthesis of porous ZnO nanoparticles using zinc nitrate [Zn(NO_3_)_2_] and sodium oxalate (Na_2_C_2_O_4_) and alkali lignin–amine as a template revealed that the ions dissociated from the lignin amine template during the reaction and, attractive electrostatic forces between Zn^2+^ and C_2_O_4_^2−^ develop. The hydrogen bonding and van der Waal forces of attraction responsible for the direct contact between the ZnO particles has been reduced through steric hindrance caused by lignin amine molecule as template, and thus resulted in lower aggregation and generation of smaller-size particles (average of 15–44 nm) [18]. According to the findings, the native lignin is deficient in positively charged functional groups, leading to poor interaction with ZnO particles [18, 19]. Introduction of positively charged moieties to the alkali lignin through the process of quaternization developed strong affinity with the negatively charged ZnO nanoparticles, which was favorable for the fabrication of lignin–ZnO composite [18].

In another work, lignin–phosphate quaternary ammonium salt (LPQAS) was formed from modifying alkali lignin through a Mannich reaction. LQPAS has been applied as a surfactant for the assembly of ZnO crystallites via precipitation with NaOH as the precipitating agent. During the synthesis, LQPAS dissociates into phosphate ions (negatively charged) and quaternary ammonium ions (positively charged). During a chemical reaction, the positively charged quaternary ammonium ions develop an interaction with the negatively charged OH^−^ ions [74]. Subsequently, LQPAS acts as a slightly negatively charged surfactant, which results in an interaction with the positively charged face [001] of ZnO particles. This interaction represents the driving force for the assembly of ZnO nanoparticles. Reducing the pH value to 7 results in the deposition of porous ZnO nanoparticles. The size of the mesoporous nanoparticles mainly depends on the ratio of Zn^2+^ and OH^−^ ions, while the surface area of the ZnO particles is mainly determined by the amount of surfactant molecules rather than the molar ratio of Zn^2+^ and OH^−^ ions [75].

Lignosulphonates are derivatives of lignin, which can be obtained through the sulfite pulping process and subsequent sulfonation, degradation, and solubilization in water [76]. The functionalization of lignin with various hydrophilic (hydroxyl, sulfonic, and carboxyl groups) and hydrophobic (aliphatic and aromatic groups) moieties results in diverse surface characteristics. Miao et al. applied sodium lignosulphonates (SLS) as template for engineering ZnO nanomaterials [77]. The reaction proceeds with the interaction of positively charged zinc ions with negatively charged hydroxyl ions, resulting in the formation of [Zn(OH)_4_]^2−^, which is transformed to Zn(OH)_2_ in an alkaline medium [74] and finally to crystalline ZnO particles [77]. Morphologically different ZnO nanomaterial is obtained depending on the aggregation behavior of SLS and the electrostatic interaction developed between the negatively charged (sulfonic and carboxyl) moieties and the positively charged face [001] of ZnO crystal. Moreover, SLS adsorbed on the surface of ZnO crystallites prevents the aggregation of particles to some extent and later contributing to the self-assembly of ZnO particles in the direction of SLS produce secondary superstructures [77].

Varying the amount of SLS causes different degrees of association in the solution and thereby resulting in fabrication of different hierarchical structure of ZnO nanoparticles. Lowering the concentration of sodium lignosulphonates causes aggregation attributed to steric repulsions that result in the formation of bars of ZnO clusters. Increasing the concentration of sodium lignosulphonates forms a bi-layer structure called mesh-lamina ZnO. Further increase in concentration changes the structure from bilayer to spherical bilayer, ultimately resulting in quasi-spherical particles of ZnO [77].

**Fig. 6:**
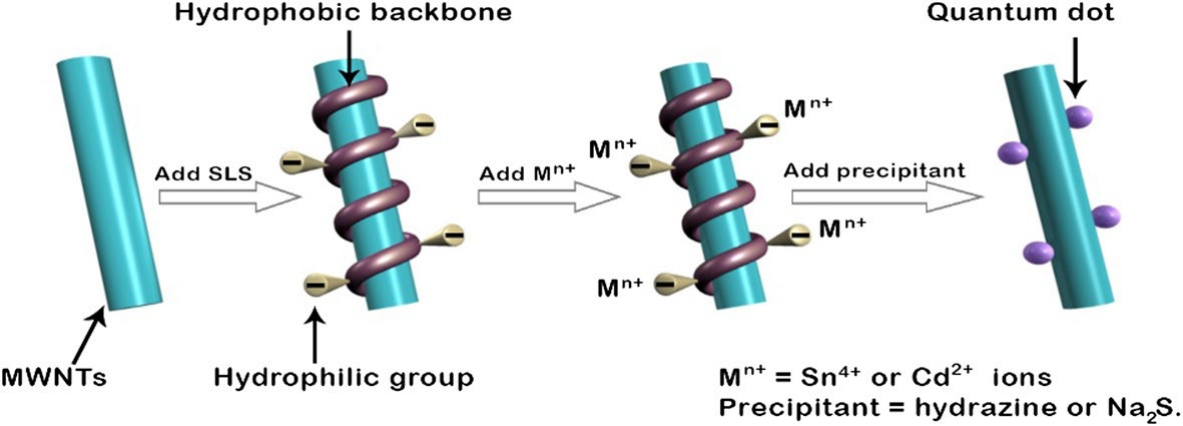
Mechanism for the Functionalization of MWNTs and quantum dot decoration [79]

SLS have also been used for the surface functionalization of multi-walled carbon nanotubes (MWNTs). Sodium lignosulphonates act as a dispersing agent and are adsorbed on the surface of the MWNTs. Π–Π non-covalent stacking is mainly responsible for interaction between SLS and MWNTs. SLS is amphiphilic in nature, but dominated by hydrophobic groups with ether and C–C bonds. Other than hydrophobic linkages, Π–Π stacking interactions are also responsible for the adsorption of sodium lignosulphonate on the MWNTs. The steric repulsion caused by sodium lignosulphonate helps in minimizing the van der Waals forces of attraction at the surface contact. The anionic groups on the surface of the sodium lignosulphonate extrude outward to reduce the electrostatic forces of repulsion. The similar charges on the sodium lignosulphonates make MWNTs extremely hydrophilic and, consequently, responsible for their solubility and stability in aqueous medium [78]. SLS-functionalized MWNTs serves as a potential support for the fabrication of quantum dot hybrids. The uniform deposition of SnO_2_ and CdS nanoparticles on the SLS-functionalized MWNTs template comprises the interaction between the positively charged ions of the nanoparticles (Sn^4+^ and Cd^2+^) and the negatively charged groups of sodium lignosulphonates, as shown in Fig. 6. The SLS-functionalized MWNTs is an outstanding support for the fabrication of quantum dot hybrids ensuring the stability of over 6 months at ambient temperature [79].

#### Applications of Lignin-Based Composites in Photocatalysis

The interest in applying lignin in material engineering is increasing [9, 80] specifically in the case of preparing composite materials for photocatalytic applications [72]. The application of lignin as photocatalyst support allows controlling the size of the particles and to obtain a uniform distribution of the particles of the photocatalyst [70]. Additionally, lignin is derived from renewable biomass resource as a byproduct from the pulp and paper industry [81]; consequently, the application of lignin in material engineering will reduce the cost as well as help to fabricate environmentally compatible composites. Hence, it is considered to be a promising support for the synthesis of composite photocatalyst. Recently, some attempts have been made to develop lignin-based photoactive composites to improve the efficiency of the photocatalytic process [18, 19]. In order to overcome the limitations of the photocatalytic reaction, there is a need to understand the basic photophysical and photochemical mechanism of the process. The interaction of photoactive material and light is an important factor in determining the efficiency of the photocatalytic reaction. Nevertheless, the redox reaction can be impeded by the large band gap between valence band and conduction band in the semiconductors [82, 83]. Therefore, high-energy UV radiation is required for carrying out the photochemical reaction, which is the principal constraint associated in upscaling of a photocatalytic conversion [82, 84].

Lignins form a dark-colored solution in most of the solvents, which makes it photocatalytically inactive, which may reduce the efficiency of the photocatalyst [85]. Nevertheless, it has been observed that lignin can be applied as template for the assembly of stable hybrid photocatalysts. The interaction between the components in the case of composites plays a significant role in determining the photocatalytic performance [86]. The properties of the composites are diverse due to the reinforcing synergy between the components of the hybrid photocatalyst. Consequently, this enhances the transfer and utilization efficacy of photogenerated electrons and intensify the separation of charges that synergistically boost photocatalysis by overcoming the chances of recombination of electrons and holes [82, 83, 86, 87]. Hence, the photocatalytic efficiency could be improved by fabricating a composite of photoactive material and appropriate support. Additionally, the chemical and physical stability of the composite photocatalyst mainly depends on the nature of the support [86]. The use of support stabilizes the textural properties upon thermal treatment, which enhances the disseminations of active sites and usually enhances the catalytic activity of pure oxides [88, 89].

Photocatalysts based on lignin semiconductor composites have great potential for the remediation of contaminated water and have received much attention in recent years [19, 72]. The use of lignin as a photocatalyst support would endorse distinctive functionality with excellent physicochemical properties for specific applications accredited to the interaction between semiconductor and support [90]. The following section provides an overview of recent applications of lignin-based composites for contaminant degradation. As reported in the recent literature, the assimilation between lignin and metal oxides, such as ZnO, TiO_2_, or CuO, increases the degradation of pollutant compared to pristine metal oxide alone [19, 72, 77].

Lignin-amine (LA) mesoporous zinc oxide hybrid catalyst depicted high sunlight photocatalytic activity. Introduction of amine groups to the lignin via amination reaction improves the surface activity as well as the flocculation and decolorization efficiency for the treatment of wastewater [81, 91]. The calcination temperature during catalyst preparation plays a significant role in determining the size, morphology, microstructure, and photocatalytic performance of the ZnO nanophotocatalysts. ZnO–LA composite calcined at 400 °C exhibits higher photocatalytic efficiency than those calcined at 500 and 600 °C. Increasing the temperature from 400 to 600 °C resulted in an increase in size and decrease in specific surface area of the photocatalyst. In addition, doping of LA with ZnO precursor also contributes to acquire smaller size and high specific surface area of ZnO nanoparticles by preventing the agglomeration of ZnO particles. ZnO–LA annealed at 400 °C exhibited photocatalytic degradation efficiency of 99.2 and 96.4% for methyl orange (20 mg l^−1^) under UV light irradiation (1 h) and under solar radiation (6 h), respectively. The solar photocatalytic performance of ZnO–LA is almost the same as that of TiO_2_ (P25) [81]. During a photochemical reaction, the water adsorbed on the surface is oxidized by holes to ^·^OH radicals, while O_2_ adsorbed on the surface of ZnO is reduced by the electrons. The hydroxyl radical and superoxide ion formed result in mineralization of methyl orange [90].

In addition to aminated lignin, sodium lingosulphonates have also been used for the fabrication of nano ZnO photocatalyst. Different ZnO morphologies such as nanoparticle-bar, nanomesh-lamina, and quasi-nanosphere were acquired [77], depending on the concentration and aggregation of sulphonated lignin and electrostatic interaction between the sulphonated lignin and ZnO crystals. The lignosulphonates–nanomesh ZnO composite displayed 100% degradation efficiency for methylene blue (5 ppm) under UV irradiation power of 12 W within 90 min (see entry 7, Table 3 for effect of irradiation power on degradation efficiency) [77]. In the course of photocatalytic degradation, holes generated react with either hydroxyl ions or adsorbed water molecules and form hydroxyl radicals. Simultaneously, the interaction of O_2_ molecules and electrons produce superoxide anion radicals. Finally, the interaction of superoxide anion radicals and hydroxyl radicals with methylene blue results in its degradation [77, 92]. Compared to pristine ZnO particles, lignosulphonate-doped ZnO particles exhibited high photocatalytic efficiency due to improve surface state, high specific surface area with more hydroxyl groups, and smaller band gap of ZnO particles [93].

**Table 3:** Applications of lignin-based composites photocatalyst in photocatalytic degradation of organic substances

Entry no.	Type of composite	Contaminant	Light source	Temperature (°C)	Photocatalytic activity (% degradation/conversion)	Reaction time (min)	References
1	Nano-ZnO–LA	Methyl orange	UV light (300 W)	25	99.20	60	[81]
			Solar radiation	28–36	96.40	360	
2	SLS-CuO/ZnO nanocomposites	Rhodamine B	Visible light	25	91.50	240	[68]
		Congo Red	500 W Xe lamp		74.30		
3	Lignin-based carbon/ZnO composite	Methyl orange Rhodamine B	Solar light (500-W Xe lamp)	15	99.90	30	[18]
					79.20	50	
4	Lignin-based carbon/ZnO	Methyl orange	Solar light (500-W Xe lamp)	15	98.90	30	[19]
	nanocomposite						
5	AL–CuO nanoparticles	Methylene blue	UV light	25	97.80	90	[70]
		Methyl orange			66.70		
6	Nano TiO_2_–lignin composite	Phenol	UV light (8-W mercury lamp)	25	97.90	120	[72]
7	SL–ZnO nanomesh lamina	Methylene blue	UV irradiation (WFH-203)				[77]
			12 W	25	100	90	
			8 W		96	120	
			5 W		88	120	
			2 W		80	120	
8	Nano ZnO–AL	Methyl orange	UV light	25	98	90	[113]

**Fig. 7:**
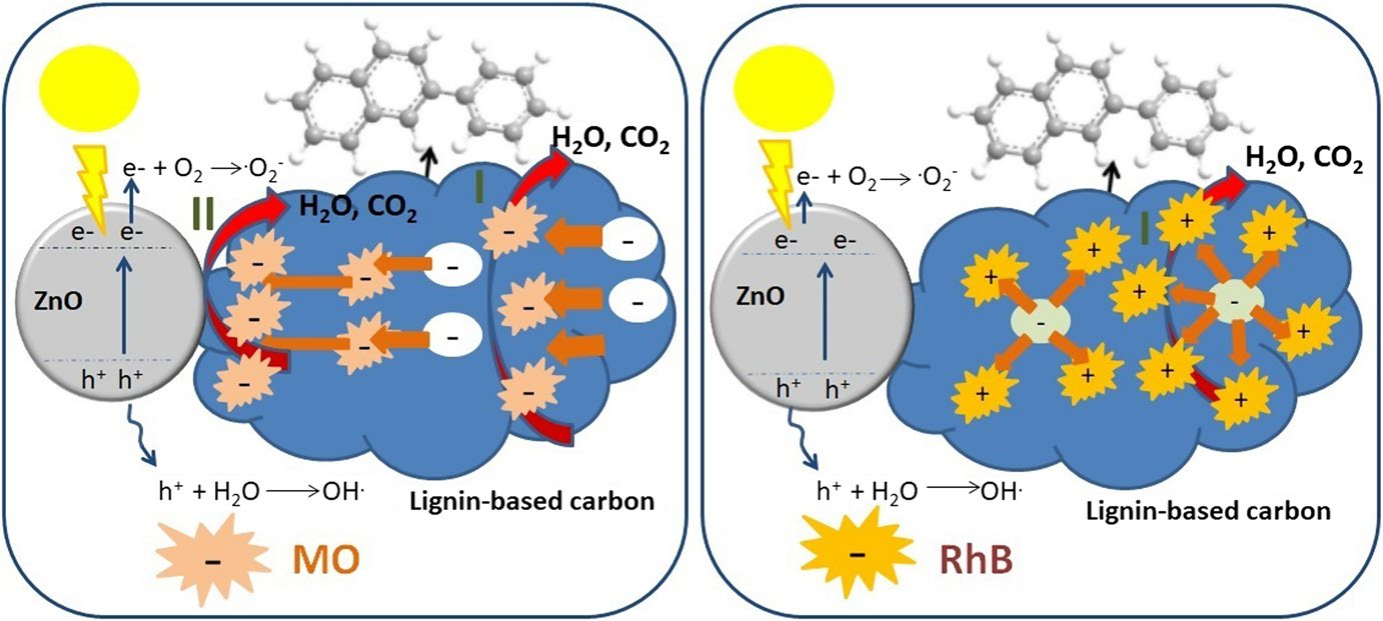
Photocatalytic mechanism for the degradation of MO (**a**) and RhB (**b**) over the LC–ZnO composite [18]

Moreover, quaternized alkali lignin–ZnO hybrid have been applied for the degradation of Rhodamine B (Rh B) and methyl orange (MO). Yet again, efficiency of composite is far better than the pure ZnO. After the light irradiation (see entry 3, Table 3 for light source details) for 30 min, methyl orange (15 mg l^−1^), was completely degraded by the composite while pure ZnO showed 75.3% degradation efficiency even after 50 min. Quaternized alkali lignin–ZnO exhibited lower degradation efficiency for 15 mg l^−1^ Rhodamine B (79.2%) compared to methyl orange but was higher than that of pure ZnO (31.1%). Methyl orange mainly degraded by holes, accredited to the negative charge of the dye pushed towards ZnO as shown in Fig. 7. Whereas, the degradation of Rhodamine B is driven by ^·^O_2_^−^ and ^·^OH radicals due to their strong oxidative abilities [18]. The applied composites were rather stable and no obvious decrease in photodegradation efficiency was observed in three successive recycling tests [18]. Thus, doping with carbon materials improved the efficiency of ZnO by inhibiting the photocorrosion of ZnO [19] and overcoming the limitation associated with the pure ZnO as photocatalyst such as charge separation and low quantum efficiency [94].

**Fig. 8:**
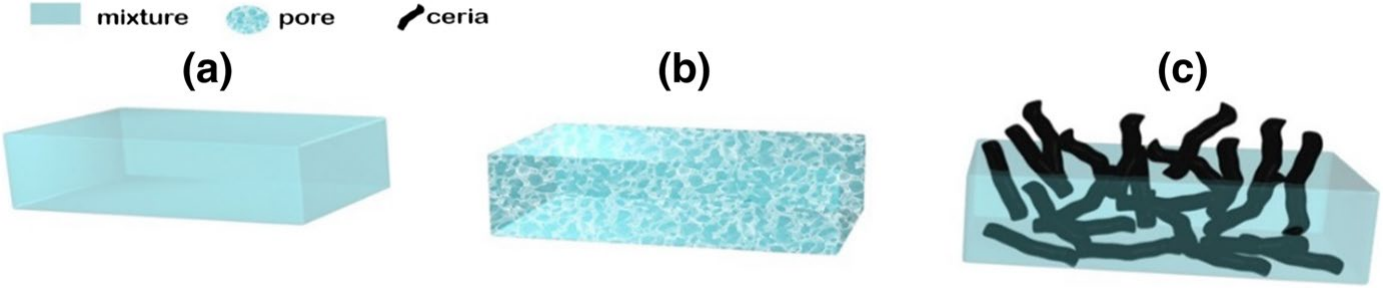
Formation mechanism of porous carbon–CeO_2_ composite. **a** Blend of lignin and cerium nitrate. **b** Partially decomposed porous lignin. **c** Porous carbon CeO_2_ composite [20]

Similarly, porous carbon-based CeO_2_ composites were fabricated applying lignin as support. Lignin decomposition contribute to the porosity of the template and ensure the uniform growth of CeO_2_ nanorods to carbon–CeO_2_ composite (Fig. 8).

The photocatalytic activity of carbon–CeO_2_ hybrid was determined for the desulfurization of SO_2_, which is extremely injurious to human health as well as for the environment. CeO_2_ plays a significant role in trapping SO_2_ molecules, oxygen storage, and oxidizing ability of CeO_2_ results in chemisorption of SO_2_. Lignin support not only takes part in physisorption but is also involved in photocatalytic conversion of SO_2_ [20, 95]. The reaction initiated with physical adsorption of SO_2_, and then chemisorption took place simultaneously due to the oxidizing and oxygen storage properties of CeO_2_. Porous carbon CeO_2_ hybrid exhibited high desulfurization efficiency and significantly oxidize the adsorbed SO_2_ with conversion ratio of 51.8%. Carbon–CeO_2_ showed improved photocatalytic performance compared to pristine CeO_2_, which is attributed to the possible involvement of carbon during a reaction.

The reaction mechanism for the desulfurization of SO_2_ is as follows (Reactions 1–3) [21]

1 $$ (1/2)x{\text{O}}_{2} + x{\text{C}} \to x{\text{C(O)}} $$

**Fig. 9:**
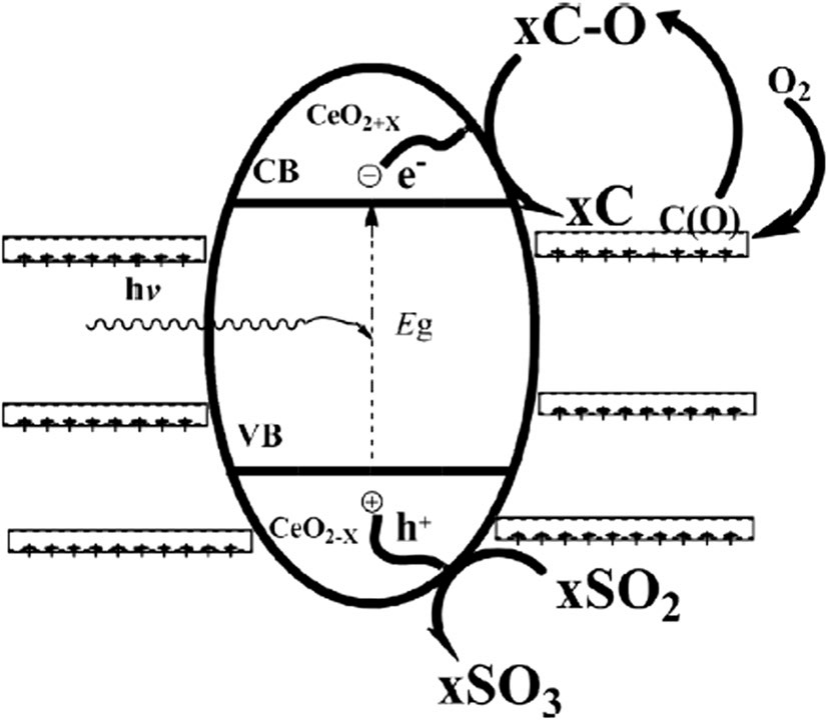
Desulfurization mechanism of porous carbon–CeO_2_ composites [20]

The reaction proceeds with the formation of active site C(O) that later is converted to carbon–oxygen complex, which provides oxygen for the photocatalytic reaction. SO_2_ reacts with the holes and O_2_ from the CeO_2_, and is thereby converted to SO_3_ (Fig. 9). The electrons on the surface of the catalyst react with the carbon–oxygen complex, simultaneously CeO_2_ retains the O_2_ and generates carbon [20].

2 $$ {\text{CeO}}_{2} + x{\text{SO}}_{2} \to x{\text{SO}}_{3} + {\text{CeO}}_{2 - x} $$

3 $$ {\text{CeO}}_{2 - x} + x{\text{C}} - {\text{O}} \to x{\text{C}} + {\text{CeO}}_{2} $$

There are very few studies reported on the utilization of lignin as support for the synthesis of mesoporous TiO_2_ photocatalyst. Chen et al. synthesized mesoporous TiO_2_ composite using TiCl_4_ as the reactant and lignin as the template [72]. The synthesized photocatalyst was used for phenol (0.05 g l^−1^) degradation under UV light, resulting in degradation of 97.9% of phenol in 120 min (entry 6, Table 3). The photocatalytic performance of TiO_2_–lignin composite was reported to be higher than the TiO_2_ synthesized without template and commercial TiO_2_ P25 that showed phenol degradation efficiency of 76.3 and 86.3%, respectively. The high photocatalytic activity of TiO_2_–lignin composite is due to the high electronegativity difference between lignin and TiO_2_ precursor that contributes to its uniform distribution for the formation of mesoporous TiO_2_ particles. The lower surface hydroxyl group on TiO_2_–lignin is another factor for the superior photocatalytic efficiency of composite that is ascribed to the stronger interaction between surface hydroxyl groups of TiO_2_ precursor and lignin hydroxyl groups during hydrolysis. Moreover, lignin also contributes to the smaller crystal size and high specific surface area that ultimately improved the photocatalytic performance of the composite [72].

Recently, cupric oxide (CuO), a* p*-type semiconductor, has gained much attention as a photocatalyst due to its narrow band gap (1.2 eV). It has been widely used as a photocatalyst [96, 97] for the degradation of pollutants [97] and for the production of H_2_ gas [98]. The size, morphology, microstructure, and photocatalytic performance of CuO nanoparticles was found to be enhanced by using aminated lignin (AL) as template. Calcination temperature and AL amount play a significant role in determining the photocatalytic activity of the CuO–AL composite. Under UV light irradiation for 90 min, CuO doped with AL (0.5 g) exhibited considerably higher photodegradation of 10 mg l^−1^ methylene blue (97.8%) and 10 mg l^−1^ methyl orange (66.7%) compared to undoped CuO. The optimum calcination temperature for CuO catalyst was 400 °C, which yields smaller crystallite size and high surface area, thus improving the photodegradation rate of organic dyes. Low temperature (300 °C) leads to incomplete decomposition of aminated lignin while high temperature (500 °C) resulted in aggregation of particles. The optimum dosage of aminated lignin for CuO doping was 0.5 g; further increase in dosage resulted in larger crystal size due to aggregation [70]. Moreover, optimizing the dosage and calcination temperature, the photocatalytic performance of the lignin-based composites can be maximized, in agreement with other studies [81].

### Applications of Lignin-Based Materials in Photovoltaics

Appropriate engineering of photovoltaic cells is required for optimum light harvesting capacity and improved photo-inductive charge transfer [99]. The interface engineering of the anode is of utmost significance to improve the efficiency of the cell [100]. A power conversion efficiency of 10% has been achieved through cathode modification. However, in case of the anode, water-soluble conductive polymer poly(3,4-ethylene dioxythiophene):poly(styrene sulfonic acid) (PEDOT:PSS) has been widely used. The efficiency of the PEDOT:PSS principally driven by the conductivity and highest occupied molecular orbital (HOMO) energy level. In order to control HOMO energy level, perfluorinated ionomer (PFI) was applied as dopant for PEDOT, which exhibited better efficiency than PEDOT:PSS [101]. Similarly, the use of PSS as dopant also improved the conductivity of PEDOT [102]. Nevertheless, the microstructural and electrical inhomogeneities caused by PSS due to its non-conjugated structure configure PEDOT:PSS unsuitable for hole injection layer (HIL) [103].

The development of hole transport materials based on biomaterials are of great interest in the field of organic electronic devices. Lignin and its derivatives contain several aromatic rings that strongly absorb in the ultraviolet range of the electromagnetic spectrum [104]. In organic electronic devices, hole transport process is associated with the oxidation of electron-rich compounds such as thiophene in PEDOT:PSS and carbazole in poly(vinylcarbazole). Lignosulfonate poses exceptional hole-transfer characteristics owing to oxidation of phenols and j aggregation phenomenon. The aggregation behavior is responsible for the semiconductive nature of lignosulfonates [105].

Recently, various attempts have been made to improve the conductivity of PEDOT:PSS through different additives such as ionic liquids, surfactants, and organic solvents. Post-treatment of PEDOT:PSS with inorganic acid, polar solvents, salts, and zwitterions significantly enhanced the conductivity of PEDOT [106]. To significantly improve the conductivity and reduce the production cost, there is a need for renewable dopant to boost this technology. In view of green chemistry and green economics, lignin derivatives, as an alternative semiconductive material, have been employed as dopant for PEDOT. Li et al. investigated the potential of lignosulfonate (SL) and alkyl chain cross-linked lignosulfonate polymer (ASL) obtained by the introduction of alkyl chain to sulfomethylated lignin, as hole transport material for solar cells [105]. The mass ratio of PEDOT:SL has not shown considerable change in PCEs of the cell (see entry 2, Table 4 for detailed photovoltaic performance), with PEDOT:SL mass ratio of 1:1, 1:2, and 1:3, PCEs exhibited by the device (ITO/HTM/PTB7:PC_71_BM/Al) were 5.79, 5.76, and 5.33%, respectively [105]. The PCE of PEDOT:SL is quite close to PEDOT:PSS (4.28 and 4.50%) as reported in the literature [107, 108]. Moreover, the results of PCEs of PEDOT:ASL with mass ratios of 1:1 and 1:2 were 2.14 and 3.93%, respectively. The results obtained depicted the positive influence of phenolic groups in lignosulfonates on the hole transport features of the materials. The hole mobility of lignosulfonate polymer is comparatively lower than lignosulfonate due to the reduced content of phenolic hydroxyl groups. The surface and nanoaggregate size of the film also contribute to the hole transport properties of the materials. The unique surface and variable size of nanoaggregates among PEDOT:SL and PEDOT:ASL films leads to the different hole transport properties of both materials [105].

**Table 4:** Photovoltaic performances of PSCs with different proportions of different lignin-derived dopants for PEDOT

Entry no.	Anode	Device architecture	*V*_OC_ (V)	*J*_SC_ (mA cm^−2^)	FF (%)	PCE (%)	References
1	PEDOT:PSS	HTL/CH_3_NH_3_PbI_3_/PC_61_BM/Al	1.02	18.12	68.5	12.62	[103]
	PEDOT:GSL		0.98	19.21	74.7	14.10	
	PEDOT:GSLa reverse		1.031	20.1	72.08	14.94	
	PEDOT:GSLa forward		1.026	19.93	72.45	14.82	
2	PEDOT:PSS	ITO/HTM/PTB7:PC_71_BM/Al	0.73	12.11	65.57	5.80	[106]
	PEDOT:ASL-1: 1		0.44	10.34	47.01	2.14	
	PEDOT:ASL-1: 2		0.59	12.47	53.39	3.93	
	PEDOT:SL-1: 1		0.70	13.22	62.60	5.79	
	PEDOT:SL-1: 2		0.69	13.27	62.96	5.76	
	PEDOT:SL-1: 6		0.68	12.64	62.03	5.33	
3	PEDOT:PSS	ITO/HTL/PTB7-Th:PC_71_BM/PFN/Al	0.77	15.82	68.71	8.39	[22]
	PEDOT:GSL-1: 1		0.73	16.29	63.85	7.57	
	PEDOT:GSL-1: 2		0.73	16.27	64.81	7.74	
	PEDOT: GSL-1: 4		0.77	16.25	68.17	8.47	
	PEDOT: GSL-1: 6		0.77	16.29	67.04	8.37	
4	PEDOT:SL-1: 1	ITO/HEL/PTB7:PC_71_BM/Al	0.58	15.06	58.87	5.19	[109]
	PEDOT:SL-1: 2		0.65	13.43	57.55	5.02	
	PEDOT:ASL1-1: 1		0.57	14.69	56.40	4.75	
	PEDOT:ASL1-1: 2		0.56	12.20	54.55	3.73	
	PEDOT:ASL2-1: 1		0.68	11.17	45.88	3.49	
	PEDOT:ASL3-1: 1		0.68	10.96	41.74	3.11	

Wu et al. reported the application of grafted sulfonated acetone–formaldehyde lignin (GSL), as a* p*-type semiconductive dopant for hole extracting layer [103]. GSL is a polymeric semiconductor derived by grafting the sulfonated acetone–formaldehyde (SAF) to alkali lignin (AL). The long aliphatic chain and large number of sulfonic groups on GSL make it a fine dispersant for being used as dopant for PEDOT. The conjugated structure of GSL makes it a good candidate for electron–hole mobility similar to other conjugated polymers used in organic electronics. GSL as hole transporting layer has shown promising results with the hole mobility of 2.27 × 10^−6^ cm^2^ V^−1^ s^−1^ attributed to large number of hydroxyl moieties. Furthermore, GSL:PEDOT exhibited better conductivity and power conversion efficiency up to 14.94% than PEDOT:PSS (12.6%) with the device structure of HTL/CH_3_NH_3_PbI_3_/PC_61_BM/Al. The high efficiency of PEDOT:GSL is credited to the homogeneity and uniformity of the film surface, which is instigated by highly disperse GSL. Altogether, it will improve performance of the device by increasing charge transfer properties [103]. Furthermore, larger grain size of the PEDOT:GSL film results in higher current density [49]. Indium tin oxide (ITO) modified by PEDOT:GSL exhibited larger grain size (67 nm) than ITO transformed by PEDOT:PSS (61 nm). Consequently, PEDOT:GSL modified ITO as hole-extraction layer has better transport characteristics for hole collection due to its conjugated structure than PEDOT:PSS that lacks a conjugated structure [103].

SL and ASL have exceptional properties of forming Block-like self-assembly without any external interface in particular solvents. During the oxidation of SL, characteristic aggregation behavior is acquired by SL and ASL through the electron transport mechanism and their self-assembly. SL acquires distinctive assembly, attributable to its amphiphilic nature and presence of benzene rings that leads to its aggregation in particular solvents through Π–Π interactions and CH–Π interaction. With 1:3 H_2_O: ethanol solution, the aggregates acquired for SL were of nano size, while micro-sized aggregates were obtained for ASL in the same set of conditions. Block-like aggregation behavior was more dominant in ASL compared to SL due to cross-linked alkyl chain polymerization in SL. Based on the aggregation behavior and electron transport characteristics of the SL and ASL, the materials have been applied as dopants to improve the conductivity of PEDOT [109]. The power conversion efficiency of polymer solar cell also depends on the aggregation behavior of the dopants that is ultimately affected by the hydroxyl group content [105, 109]. Moreover, the oxidative capacity of SL is much better than ASL due to the high phenolic hydroxyl group content. The reaction proceeds with the formation of radical cations and phenol radicals, formed by the oxidation of SL and phenolic hydroxyl groups, respectively. With ITO/HEL/PTB7:PC_71_BM/Al device structure, the maximum PCE showed by PEDOT:SL with mass ratio of 1:1 was 5.19% that shows the potential of SL as effective dopant for PEDOT in organic electronic devices. SL exhibited the hole mobility of 2.95 × 10^−6^ cm^2^ V^−1^ s^−1^, which is higher in comparison to ASL that showed the hole mobility of 3.18 × 10^−7^ cm^2^ V^−1^ s^−1^. The results of the study also showed that hydroxyl group content is directly related to the hole mobility and PCE, whereas increase in hydroxyl group increased hole transport ability and PCEs and vice versa. Furthermore, the high pH of SL and ASL is an advantage of the conductive polymers over conventional dopant PSS that will prevent corrosion of ITO layers [109].

Hong et al. also investigated the GSL as potential dopant and stabilizer for PEDOT to enhance the performance of light-emitting and photovoltaic devices [22]. PEDOT:GSL films and aqueous dispersions with adjustable conductivities and work functions have been used for fabricating high-performance organic light-emitting diodes and polymer solar cells [22]. GSL has a number of advantages over other lignin-derived polymers such as lignosulfonates applied as dopant for PEDOT. GSL has high phenolic content that results in better oxidative capability of the polymer. The high degree of sulfonic group in GSL compared to lignosulfonate makes it a more suitable dispersant for excellent PEDOT dispersion. The addition of GSL in PEDOT also results in better film characteristics in comparison to PEDOT modified by lignosulfonate attributed to the superior dispersing characteristics of GSL. Altogether, the superior GSL contribute in improving the hole transport properties of PEDOT as a dopant. The oxidation peak of GSL-doped electrode obtained at 1.1 V that indicates that GSL HOMO energy level is − 5.5 eV and its oxidation can take place at comparatively low potential. GSL as hole transporting material exhibited a good hole mobility (2.27 × 10^−6^ cm^2^ V^−1^ s^−1^) credited to its phenolic structure. The power efficiency of PEDOT:GSL with the mass ratios of 1:1, 1:2, 1:4, and 1:6 were 1.51 , 6.04 , 12.91, and 14.67 lmW^−1^, respectively. It is evident by the power efficiencies that by increasing the GSL content the hole injection and transport properties were improved. Similarly, PEDOT:GSL 1:4 and 1:6 displayed far better power efficiency than PEDOT:PSS (8.25l mW^−1^). Using the device structure of ITO/HTL/PTB7-Th:PC_71_BM/PFN/Al, the PCEs of PEDOT:GSL with mass ratios of 1:4 and 1:6 was 8.47 and 8.37%, respectively, which is quite analogous to PEDOT:PSS (8.39%) (entry 3, Table 4). Moreover, the homogenous film surface of PEDOT:GSL results in enhancement of hole injection features of the material due to the excellent dispersion property of GSL. Another important function for organic electronic devices is work function that also increases with increasing GSL content. The work function of PEDOT:GSL with mass ratios of 1:2, 1:4, and 1:6 were 4.92, 5.05, and 5.10 eV, respectively, which is comparable to PEDOT:PSS (5.02 eV) [22].

## Conclusions, Future Perspectives, and Challenges

In this review, we discussed the recent progress in the field of photocatalysis and photovoltaics with a focus on lignin-based composite materials. The aims were to review the recent studies on the application of lignin-based materials for photocatalysis and photovoltaics-related environmental remediation and energy conversion, respectively, which will provide some useful implications for future research. Lignin as a biopolymer support showed promising potential in the field of heterogeneous photocatalysis explicitly in the context of the degradation of unwanted contaminants in the environment. A number of studies have reported the use of lignin as a commendable template for photocatalyst synthesis [18] attributed to its high specific surface area that improves the physical adsorption of the substrate [20] and superior photoelectron transfer characteristics owing to unique surface contact [18]. In addition, lignin-based composite materials have great potential to replace cost-intensive materials like graphene in the field of photocatalysis. The photodegradation efficiency of graphene oxide-based composites for methyl orange, methylene blue, and Rhodamine B were 87.2, 85.1, and 73.9%, after 60 min of UV photodegradation, respectively [110]. Lignin composite possesses excellent photocatalytic activity (see Table 3 for details), which is superior to those of graphene.

Many methods are suitable for the preparation of lignin-based composites. However, pH-assisted precipitation and solid-phase grinding have attracted increasing attention and some promising results have been reported. In future research on the photocatalysis via lignin-based composites, detailed investigations on the interaction of composite and substrate should be performed. Moreover, prospective applications may be expected in the field of photoelectric conversion [19], and for electrochemical storage systems [42], such as supercapacitors [15].

There are a number of studies discussed in this review demonstrating that lignin-based materials have wide applications in diverse fields ranging from photocatalysis to electrochemical energy devices and biomedicine. Developing lignin into functional materials, specifically its application as a support for solid composite photocatalysts, would present a great success. Moreover, lignin-based composites show improved photocatalytic efficiency, e.g., for the degradation of pollutants in aqueous media. Nevertheless, a deeper understanding of the underlying mechanism of interaction of lignin and the photoactive material are in demand.

Although considerable advancement has been made in materials development and understanding the structure–property relationships of organic photovoltaics materials and devices, there are still numerous open questions that need to be answered to achieve an increase in the photoconversion efficiency in order to reach at an economically feasible utilization. Particularly, the charge-carrier separation and mobility within the materials has to be improved. There are very few studies that have explored the potential of lignin as dopant for anode in photovoltaic devices. However, recent success in development of lignosulfonate-based dopants for semiconductive polymers with PCE analogous to PSS (see Table 4 for comparison) will pave the way for future research in lignin-based high-performance organic devices. Further research is required to understand the origin of such substantial electron transport properties of lignin as well as the mechanism of the lignin-based photovoltaic cells with improved performance.

The valorization of lignin not only encompasses technical and scientific developments but also economic aspects. There are striking opportunities for an economic gain from lignin valorization attributed to its low cost and profuse availability as a byproduct of the pulping industry and bioethanol refineries. Hence, the potential to apply the underutilized lignin sources stimulates the aspiration not only for the development of efficient isolation methods but also for the fabrication of new lignin-based products, which have high economic value in the coming years.

## Abbreviations

UV: Ultraviolet
*T*_g_: Glass transition temperature
*T*_d_: Degradation temperature
RhB: Rhodamine B
ITO: Indium–tin-oxide
HOMO: Highest occupied molecular orbital
LUMO: Lowest unoccupied molecular orbital
PEDOT:PSS: Poly(3,4-ethylenedioxythiophene): poly(styrene sulfonate)
P3HT: Poly(3-hexylthiophene)
PCBM: [6]-Phenyl-C61-butyric acid methyl ester
LA: Lignin-amine
MO: Methyl orange
AL: Aminated lignin
LPQAS: Lignin-phosphate quaternary ammonium salt
SLS: Sodium lignosulphonates
PFI: Perfluorinated ionomer
HIL: Hole injection layer
SL: Lignosulfonate
ASL: Alkyl chain cross-linked lignosulfonate
PCE: Photoconversion efficiency
GSL: Grafted sulfonated acetone–formaldehyde lignin
G: Guaiacyl
S: Syringyl
H: *p*-Hydroxybenzaldehyde
SAF: Sulfonated acetone–formaldehyde
MWNTs: Multi-walled carbon nanotubes
AL: Alkali lignin
HTL: Hole transport layer
HTM: Hole transport material
HEL: Hole extracting layer
PC_71_BM: [6,6]-Phenyl-C_71_-butyric acid methyl ester
PTB7: Poly[[4,8-bis[(2-ethylhexyl)oxy]benzo[1,2-b:4,5-*b*′]dithiophene-2,6-diyl][3-fluoro-2-[(2-ethylhexyl)carbonyl]thieno[3,4-*b*]thiophenediyl]]
Th: Benzodithiophene
J_SC_: Short-circuit current density
*V*_OC_: Open-circuit voltage
FF: Fill factor
PFN: Poly[(9,9-bis(3′-(*N*,*N*-dimethylamino)propyl)-2,7-fluorene)-alt-2,7-(9,9-dioctylfluorene)]
[Ir(ppy)2(dtbbpy)]PF6: 4,4′-Di-*tert*-butyl-2,2′-bipyridine)bis[(2-pyridinyl)phenyl]iridium(III) hexafluorophosphate
